# The microRNA-200/Zeb1 axis regulates ECM-dependent β1-integrin/FAK signaling, cancer cell invasion and metastasis through CRKL

**DOI:** 10.1038/srep18652

**Published:** 2016-01-05

**Authors:** Christin Ungewiss, Zain H. Rizvi, Jonathon D. Roybal, David H. Peng, Kathryn A. Gold, Dong-Hoon Shin, Chad J. Creighton, Don L. Gibbons

**Affiliations:** 1 Department of Thoracic/Head and Neck Medical Oncology, The University of Texas MD Anderson Cancer Center, Houston, TX 77030, USA; 2 Department of Molecular and Cellular Oncology, The University of Texas MD Anderson Cancer Center, Houston, TX 77030, USA; 3 Department of Bioinformatics and Computational Biology, 1400 Pressler St., The University of Texas MD Anderson Cancer Center, Houston, TX 77030.; 4 Department of Medicine and Dan L. Duncan Cancer Center, Baylor College of Medicine, Houston, TX 77030.

## Abstract

Tumor cell metastasis is a complex process that has been mechanistically linked to the epithelial-mesenchymal transition (EMT). The double-negative feedback loop between the microRNA-200 family and the Zeb1 transcriptional repressor is a master EMT regulator, but there is incomplete understanding of how miR-200 suppresses invasion. Our recent efforts have focused on the tumor cell-matrix interactions essential to tumor cell activation. Herein we utilized both our Kras/p53 mutant mouse model and human lung cancer cell lines to demonstrate that upon miR-200 loss integrin β1-collagen I interactions drive 3D *in vitro* migration/invasion and *in vivo* metastases. Zeb1-dependent EMT enhances tumor cell responsiveness to the ECM composition and activates FAK/Src pathway signaling by de-repression of the direct miR-200 target, CRKL. We demonstrate that CRKL serves as an adaptor molecule to facilitate focal adhesion formation, mediates outside-in signaling through Itgβ1 to drive cell invasion, and inside-out signaling that maintains tumor cell-matrix contacts required for cell invasion. Importantly, *CRKL* levels in pan-cancer TCGA analyses were predictive of survival and CRKL knockdown suppressed experimental metastases *in vivo* without affecting primary tumor growth. Our findings highlight the critical ECM-tumor cell interactions regulated by miR-200/Zeb1-dependent EMT that activate intracellular signaling pathways responsible for tumor cell invasion and metastasis.

Lung cancer is the leading cause of cancer-related death, primarily due to the development of invasive and metastatic disease^1^. Approximately two-thirds of patients are diagnosed with advanced disease, and ~50% of early-stage patients recur after surgical resection. This biology underscores the need for a better understanding of the processes driving metastasis. Work by our group identified a mutant p53 allele (p53^R172HΔG^) that confers metastatic potential to lung adenocarcinomas arising in genetically-engineered mice due to a latent, somatically-activated Kras^G12D^ allele (Kras^LA1^)^2^. Comparative mRNA profiling of the primary and metastatic tumors from this model revealed a metastasis signature of differentially expressed genes that stratified a subset of lung cancer patients with poor prognosis^3^. These findings demonstrate that the *Kras*^*G12D*^; *p53*^*R172HΔG*^ (KP) mice recapitulate genetic and clinical features of metastatic lung adenocarcinoma and provide a useful model to study the mechanisms of tumor progression and metastasis.

Epithelial tumor cells can acquire the ability to invade and disseminate by undergoing an epithelial-mesenchymal transition (EMT), a developmental program that facilitates migration due to the loss of cell-cell attachments, a shift from apical-basal polarity to front-rear polarization, and appearance of mesenchymal characteristics^4^,^5^. The two-handed zinc-finger δEF1 family factors ZEB1 and ZEB2 are among several transcriptional repressor families that induce EMT^4^,^6^,^7^,^8^ by suppression of E-cadherin and other epithelial differentiation genes, upon binding to E-boxes in their promoters^9^. MicroRNAs (miRs) are small non-coding RNAs that control development and maintenance by pleiotropic regulation of cellular functions^10^. The five members of the microRNA-200 family (miR-141, −200a–c, −429) are expressed broadly in epithelial cells^11^. Expression during lung development begins in the pseudoglandular phase and is maintained in maturity^12^. In normal and cancerous epithelial cells the miR-200 family exists in a double-negative feedback loop with the ZEB1/2 transcriptional repressors^13^,^14^,^15^,^16^,^17^. The ZEB1/miR-200 balance is regulated by EMT inducers such as TGFβ^14^,^17^, which lead to loss of miR-200 expression and a shift to a mesenchymal state. Along with EMT, expression of ZEB1 and loss of miR-200 has been linked to the development of stem-like features and chemoresistance^18^,^19^. Evidence from several tumor types, including breast, ovarian and lung, implicates miR-200 repression as a prognostic or predictive factor^14^,^16^,^20^,^21^,^22^.

Although it has been shown that miRNA-200 loss is necessary and sufficient to drive EMT, the specific targets accounting for the invasive and metastatic phenotype are incompletely understood. Actin cytoskeletal reorganization is a characteristic alteration that drives cellular morphologic changes that facilitate migration, invasion and recruitment of metalloproteases necessary for extracellular matrix (ECM) degradation. Our prior proteomic profiling demonstrated that the miR-200/Zeb1 axis simultaneously regulates tumor cell-intrinsic features and the extracellular matrix composition to alter cell-matrix interactions^23^. Given that cell-intrinsic EMT is insufficient to produce invasion of tumor cells in 3D cultures with defined synthetic matrices^24^ and that *in vivo* metastasis is driven by a subpopulation of mesenchymal cells located at the tumor-stromal interface^25^, we sought to define the cell-matrix interactions facilitating invasion and metastasis with our combined *in vitro*/*in vivo* system of metastatic lung adenocarcinoma.

The heterodimeric integrins serve as mechano-signaling receptors, coupling ECM ligand and stiffness changes to intracellular signaling pathways. Integrin signaling involves the activation of the focal adhesion kinase (FAK), leading to adaptor molecule recruitment (e.g. paxillin and p130Cas). Adaptor molecule binding facilitates focal adhesion complex formation and activates downstream signaling pathways to couple cell-matrix interactions to cytoskeletal reorganization. CRKL is an adaptor molecule in the CRK protein family that is known to directly interact with paxillin and p130Cas and relocates to sites of integrin-mediated focal adhesion formation^26^. It is overexpressed or amplified in many cancer types, including lung, breast, ovarian and colon, and has been implicated in proliferation, adhesion, survival, migration and invasion^27^,^28^,^29^.

Herein we demonstrate that miR-200 loss dramatically sensitizes tumor cells to secondary activation by the ECM and investigated how the miR-200/Zeb1 axis controls the matrix-dependent tumor cell invasion and metastasis. Using our well-defined KP tumor cell model and human NSCLC cell lines in 2D/3D tissue culture models and *in vivo* studies, we found that integrin β1-collagen I interaction drives ECM-mediated FAK signaling and is required for Zeb1-dependent EMT, invasion and metastasis. Direct regulation of CRKL by miR-200 modulates focal adhesion formation and FAK/Src complex assembly to specifically mediate the cytoskeletal reorganization, formation of pro-invasive structures and *in vivo* metastasis. Data from multiple large tumor sets from The Cancer Genome Atlas (TCGA) reveals that *CRKL* expression is negatively correlated with miR-200 family expression, positively associated with *ZEB1*, EMT status, and Src signaling. Higher *CRKL* expression is associated with poor patient outcome across multiple tumor types. These findings elucidate a specific miR-200 target responsible for regulating the sensitivity of tumor cells to integrin β1-collagen I activation by the surrounding ECM, which defines both their invasive and metastatic phenotype.

## Results

### Zeb1/miR-200 balance induces a functional EMT in non-invasive epithelial tumor cells

**Figure 1:**
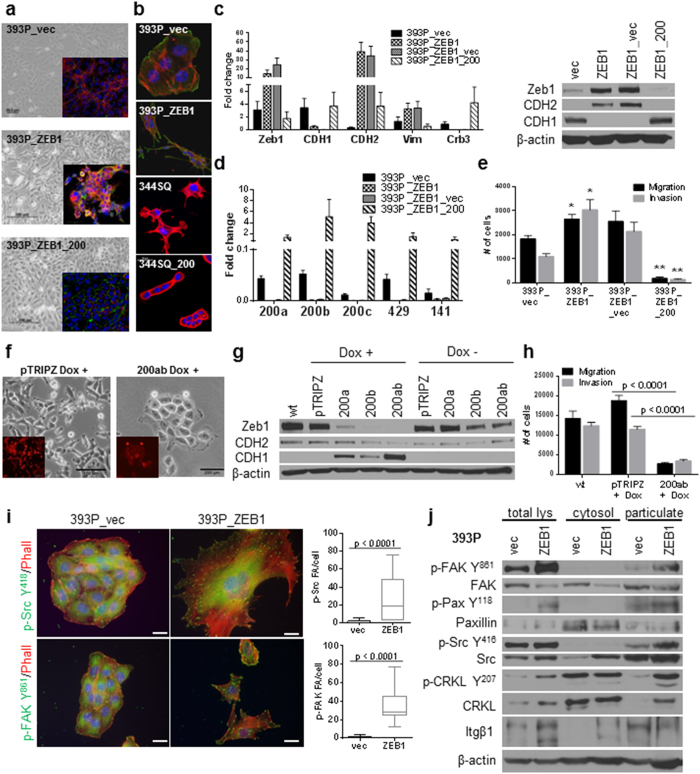
Zeb1 expression induces EMT/miR-200 repression and FAK pathway activation in previously non-invasive cells. (**a**) Zeb1 expression causes a morphology change (middle panel) and is reversed upon re-expression of miR-200 (lower panel). Cells were stained with Integrin α6 (red), ZO-1 (green) and DAPI. (**b**) 393P cells with constitutive Zeb1 expression and 344SQ cells with constitutive miR-200 family expression grown on agarose and stained with phalloidin (red), DAPI and cortactin (green, lower panels). (**c**) Quantitative RT-PCR (left) and Western blot (right) analysis of EMT markers in the indicated cell lines. (**d**) Taqman RT-PCR for the miR-200 family members in the indicated cell lines. (**e**) *In vitro* Transwell migration and invasion assay for the 393P cell line panel. *p < 0.004, **p < 0.001 (**f**) Induction of miR-200a and b in H157 causes a morphology change. (**g**) Western Blot analysis of EMT markers in H157 + /− miR-200a and/or b. (**h**) *In vitro* Transwell migration and invasion assay for the inducible H157 cells. (**i**) Immunofluorescence of 393P_vec and 393P_ZEB1 cells stained for p-Src Y^418^, p-FAK Y^861^, and phalloidin (left); an average of 15–30 cells was counted for focal adhesions/cell with individual puncta considered a focal adhesion. Scale bar is 200 μm. **(J)** Western blot analysis of total cell lysates, cytosolic or membrane particulate fractions from 393P_vec and 393P_ZEB1 cells.

Our previous work with metastasis-prone tumor cells from the KP murine model revealed that miR-200 repression is necessary in a subset of cells at the tumor periphery to produce EMT, invasion and distant metastasis^30^,^31^. To study the effects of Zeb1 expression in tumorigenic, but non-metastatic cells (393P) and compare the observed phenotype to that of metastasis-competent cells with high endogenous Zeb1 levels (344SQ), stable transfectants were generated with constitutive Zeb1 expression. Constitutive Zeb1 expression in the epithelial 393P cells from the KP model produced a dramatic morphologic change, with cells displaying a scattered, disorganized, spindle-like, fibroblastic shape (Fig. 1a,b). This change was partially reversed by re-expression of miR-200, which restored cell-cell junctions (lower panel of Fig. 1a). Immunofluorescent staining demonstrated that the mesenchymal, invasive 344SQ and 393P_ZEB1 cells display extended cell protrusions, long actin stress fibers and thin cell bodies with enhanced cell matrix contact, while miR-200 expression produced rounded, clustered cells with cortical actin staining and minimal matrix contact (393P_vec or 344SQ_miR-200) (Fig. 1b). The morphologic changes observed with Zeb1 expression were concordant with an EMT, as judged by the mRNA and protein levels of epithelial and mesenchymal markers (Fig. 1c) and decreases in the miR-200 family members (Fig. 1d). As a functional consequence, *in vitro* migration and invasion were enhanced in the previously non-invasive 393P cells, and suppressed upon miR-200 re-expression (Fig. 1e). Similar results were obtained with the mesenchymal human H157 lung cancer cell line with a stable doxycycline-inducible system to express miR-200a and/or b at levels ~20-fold baseline^32^, which produced an MET with morphologic reorganization into tight epithelial clusters (Fig. 1f), suppression of ZEB1 and other mesenchymal markers, and re-expression of epithelial protein markers (Fig. 1g). The most pronounced effect was seen upon combined miR-200a and b expression. This MET with expression of miR-200 significantly suppressed the migratory and invasive ability of the H157 cells (Fig. 1h).

### Zeb1-induced EMT enhances focal adhesion formation and signaling

To further investigate the morphologic changes associated with Zeb1 expression, cells were stained for activated Src (p-Src Y^418^) and FAK (p-FAK Y^861^). Co-staining with phalloidin and analysis of the immunofluorescent images demonstrated that Zeb1 expression induces the development of distinct puncta marked by activated Src and FAK (Fig. 1i), consistent with a marked increase in focal adhesion formation along the cell periphery and anchoring the extensive mesenchymal cell protrusions. We observed increased activation of FAK (Y^861^), Src (Y^416^), paxillin (Y^118^) and CRKL (Y^207^) in the total cell lysates, and biochemical fractionation of vector or Zeb1-expressing cell lysates into the cytosolic and particulate membrane fractions revealed increased localization of these activated proteins in the particulate fraction of the mesenchymal cells (Fig. 1j), despite equivalent recovery of the membrane fraction by comparing Itgβ1 levels. These data demonstrate that Zeb1-dependent EMT enhances membrane localization and activation of the proteins involved in FA formation.

### miR-200 repression alters collagen I-dependent cell-matrix interactions

**Figure 2:**
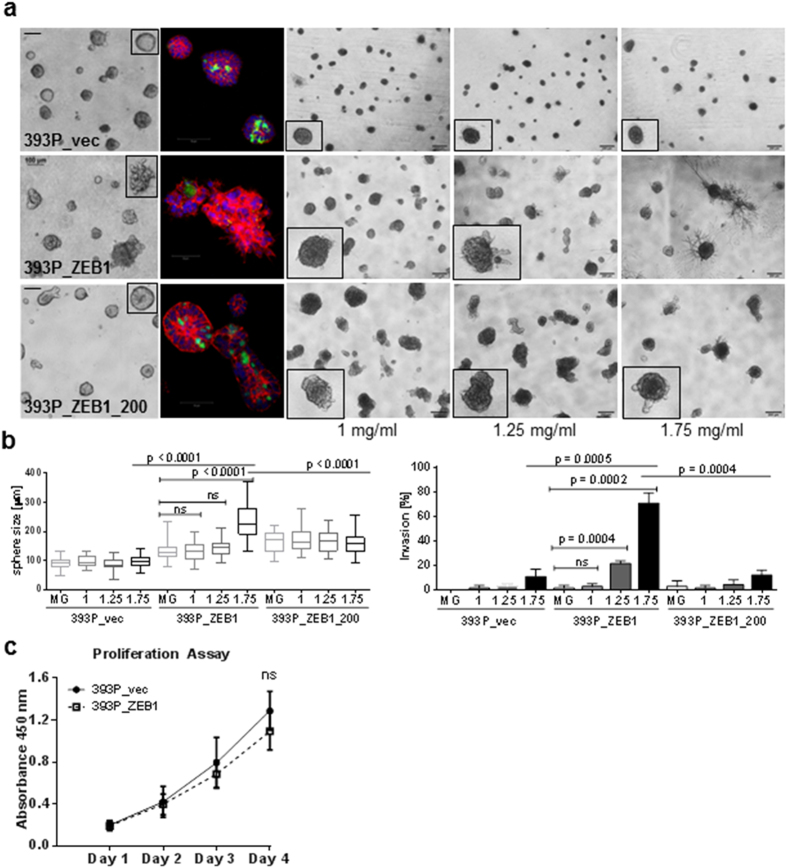
miR-200 repression potentiates cells to collagen I-dependent interactions. (**a**) Morphology of spheres grown in Matrigel (left two columns) and stained with integrin α6 (red), ZO-1 (green) and DAPI; or grown in a mixture of Matrigel and collagen I at the indicated concentrations. (**b**) Quantification of the spheres grown in Matrigel (MG) and Matrigel/Collagen at day 7. For each condition 30 structures were measured in size and 75 structures scored for invasive protrusions. (**c**) Proliferation of 393P_vec vs. 393P_ZEB1 cells over a time period of 4 days.

Given the enhanced focal adhesion complex formation and signaling observed in 2D cultures, we studied the importance of the ECM in regulating tumor cell behavior in coordination with Zeb1/miR-200 expression changes in a well-established 3D culture assay^33^. To model the extracellular matrix composition found in tumors, cells were grown on a laminin-rich Matrigel matrix or a mixture of different Matrigel/collagen type I concentrations, then phenotypically scored and stained for the cellular markers to reveal the differential 3D organization. Control cells grew as rounded, non-invasive colonies in all conditions, but upon Zeb1 expression formed larger colonies that displayed a progressively protrusive, invasive-type response to increasing concentrations of collagen type I (Fig. 2a, middle row, and 2B), which was not due to an increase in the proliferation rate (Fig. 2c). At the highest collagen concentration tested (1.75 mg/ml), ~70% of the Zeb1-expressing colonies displayed the cell protrusions indicative of invasive potential. Upon re-expression of miR-200 the cells displayed a more organized epithelial acinar structure in Matrigel culture, with pronounced cortical actin staining, and suppression of the collagen-induced protrusions (Fig. 2a, bottom row). These results suggest that miR-200 repression produces a cellular EMT while also potentiating the response of the cells to external stimulation from the collagen I-containing ECM, both of which are required to produce an invasive phenotype.

### Integrin β1 is necessary for the invasion and metastasis of the murine mesenchymal cell lines

**Figure 3:**
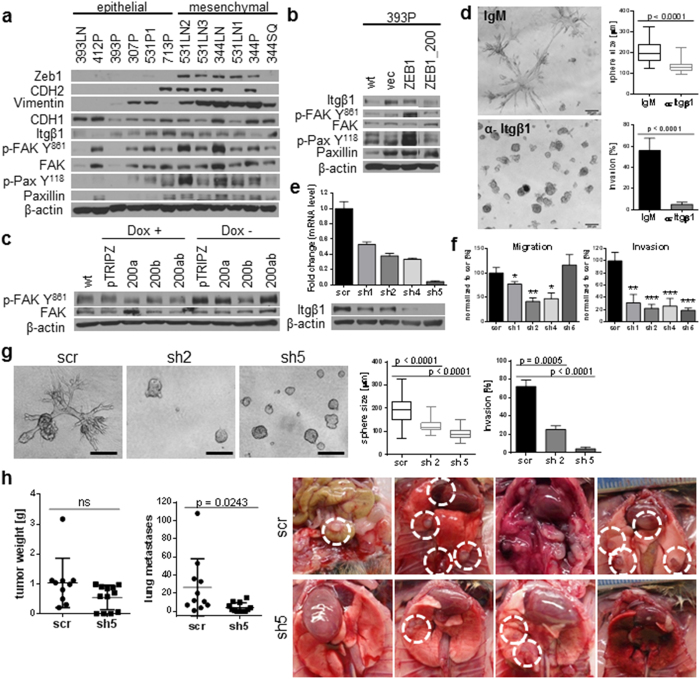
Integrin β1 is required for the invasion & metastasis of the murine mesenchymal cell lines. (**a**) Western blot analysis of FAK pathway activation in the mesenchymal vs epithelial cells lines of the murine cell line panel, and in cell lines stably expressing Zeb1 (**b**). (**c**) Western blot of FAK activation in the human H157 cells with inducible miR-200ab expression. (**d**) 393P_ZEB1 cells grown in Matrigel/Collagen I (1.75 mg/ml) and treated with an ITGβ1-blocking antibody or IgM control for 7 days. (**e**) Quantitative RT-PCR and Western blot of 393P_ZEB1 cells after ITGβ1-shRNA knockdown. (**f**) *In vitro* migration and invasion assay for the Itgβ1 shRNA cells. *p < 0.02, **p < 0.002, ***p ≤ 0.001 (**g**) 393P_ZEB1 ITGβ1 knockdown or scramble control cells grown in Matrigel/Collagen I (1.75 mg/ml) at day 6. For each condition 30 structures were measured in size and 90 structures scored for invasive protrusions. Scale bar represents 200 μm. (**h**) ITGβ1 knockdown in 344SQ cells decreases lung metastases and distant metastases *in vivo*.

Integrins are important sensors of the cellular microenvironment, transducing changes in the surrounding ECM to drive assembly of focal adhesions and downstream intracellular signaling. Screening of multiple integrin β subunits revealed that Itgβ1  mRNA expression is increased in the mesenchymal 393P_ZEB1 cells and up-regulated in the 2D vs. 3D cultures of 344SQ, paralleling the normal changes in miR-200 levels upon 3D growth (Supp Fig. S1a). However, Western blot analysis for Itgβ1 expression in a panel of KP murine cell lines stratified by epithelial or mesenchymal status demonstrated little difference in expression, which was further confirmed by FACS analysis of the cell surface expression of Itgβ1 (Supp Fig. S1g). By contrast, a significant activation of the FAK signaling pathway (including FAK and paxillin phosphorylation) was observed in the mesenchymal cells compared to the epithelial cells (Fig. 3a). Moreover, in the genetically manipulated 393P cells (Fig. 3b) and the inducible miR-200-expressing human H157 cells (Fig. 3c) we observed an inverse correlation between the activation of this pathway and the miR-200 levels, with no clear relationship to the levels of Itgβ1 or total FAK.

We next investigated the functional effect of an Itgβ1-blocking antibody on tumor cell invasion in 3D cultures. The antibody significantly decreased the observed protrusive structures of 393P_ZEB1 cells when grown in Matrigel/collagen I (Fig. 3d), as compared to an isotype-matched control antibody or a control Itgβ3-blocking antibody (Supp Fig. S1b). Additionally, combining an Itgβ3-blocking antibody with an Itgβ1-blocking antibody showed no additional effect over the Itgβ1 alone at initial plating, again demonstrating the specificity of the observation. Treatment of α-Itgβ1 at day 4, when cells had begun to display protrusive structures, still blocked the phenotype, suggesting that Itgβ1 is necessary for both the initiation and maintenance of invasive cell protrusions. To further confirm the importance of Itgβ1 in mediating matrix-dependent invasion with EMT, we used an shRNA-based knockdown approach to deplete highly invasive 393P_ZEB1 cells of Itgβ1, as confirmed by mRNA and protein levels (Fig. 3e). Knockdown of Itgβ1 significantly reduced Transwell invasion for all of the tested shRNAs, blunted migration for three of the four tested shRNAs (Fig. 3f), and produced a shift to a more epithelial morphology (Supp Fig. S1c). Furthermore, the knockdown cells displayed a decrease in activated FAK and Pax recruitment to the focal adhesion complex in the membrane (Supp Fig. S1d). Strikingly, the Itgβ1 shRNA inhibited the 3D phenotype in Matrigel/collagen I cultures of 393P_ZEB1 cells to a similar degree as the Itgβ1-blocking antibody (Fig. 3g).

To assess the functional effect of Itgβ1 on tumor cell growth and metastasis *in vivo*, we implanted syngeneic mice subcutaneously with the Itgβ1 shRNA or control scramble shRNA cells. Despite no difference in primary tumor growth, we observed a significant reduction in the number and size of distant lung metastases (Fig. 3h and Supp Fig. S1e), even when the one animal outlier was removed (Supp Fig. S1f). In addition, the control animals showed metastases in multiple distant organs, including the intestines, heart, kidney, liver and diaphragm, which were absent in the animals who received the Itgβ1 shRNA cells (Fig. 3h, photos).

### Integrin β1-collagen I contact is necessary for H157 phenotype in 3D cultures

To confirm the functional role of Itgβ1 upon EMT with human NSCLC cells, we treated H157 cells with the Itgβ1-blocking antibody either from day 0 or day 7, when the cells were starting to form protrusive structures in Matrigel-collagen I cultures. In both treatment groups, 3D invasion was significantly inhibited (Supp Fig. S2a), confirming that matrix-dependent activation was mediated by Itgβ1. Moreover, the observed effect of Itgβ1 blockade in 3D culture phenocopied the effect of inducible miR-200ab expression in the H157 cells (Supp Fig. S2f). shRNA-based knockdown of Itgβ1 decreased downstream activation of the FAK pathway (Supp Fig. S2b,c), produced a more epithelial morphology (Supp Fig. S2g) and decreased Transwell migration/invasion of H157 cells (Supp Fig. S2d). In 3D Matrigel/collagen I cultures the control cells displayed a significantly more invasive phenotype versus the Itgβ1 knockdown cells. Taken overall, these results suggest that the Itgβ1-collagen I interaction mediates pro-invasive signaling in the H157 NSCLC cells.

### CRKL is a miR-200 target that regulates integrin-dependent signaling and correlates with patient outcome

**Figure 4:**
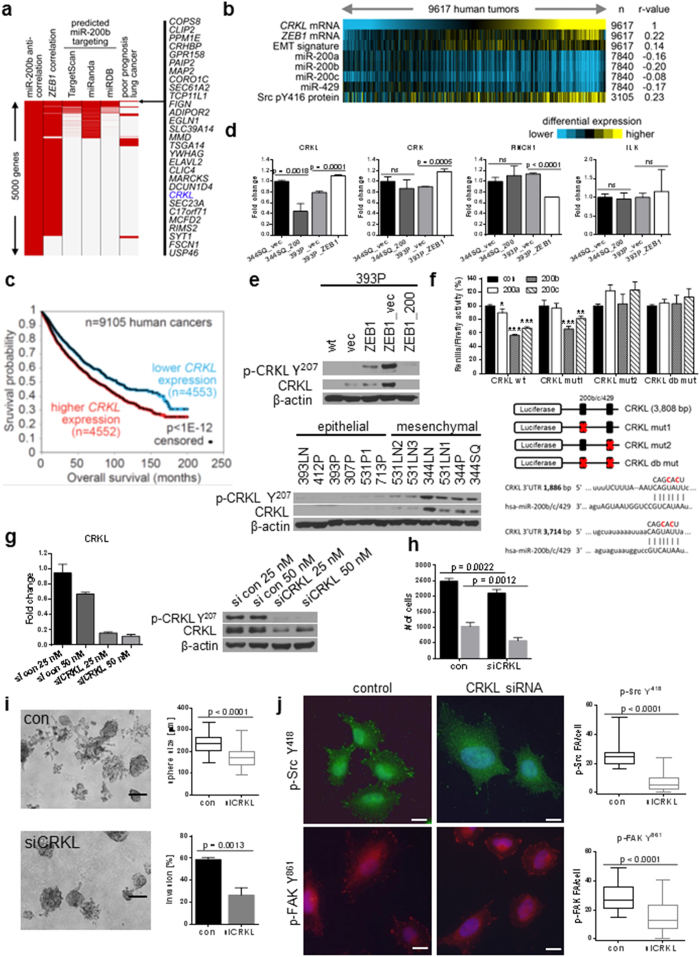
CRKL is a miR-200 target that regulates integrin-dependent signaling and is prognostic of patient outcome. (**a**) Schematic of the bioinformatic analyses applied to TCGA pan-cancer datasets to select potential miR-200b targets of relevance to lung cancer metastasis. From the top 5000 genes most anti-correlated with expression of miR-200b, we additionally considered genes positively correlated with ZEB1 expression (p < 0.0001, Pearson’s), predicted miR-200b target genes (by TargetScan, miRanda, and miRDB), and genes associated with worse prognosis (p < 0.05, univariate Cox) in lung adenocarcoinomas^25^. Genes listed met all of the above criteria. (**b**) Heatmap showing the correlation of *CRKL* expression with the listed feature for each of the tumor specimens included from TCGA pan-cancer datasets by Pearson’s coefficient. Significance of correlation: p < 1E-12 for miR-200c, p < 1E-35 all other features. (**c**) Kaplan-Meier plot of overall survival in patients from TCGA datasets, as stratified by CRKL expression. P-value by log-rank test. (**d**) qRT-PCR analysis of adaptor molecules in the paired epithelial and mesenchymal murine cell lines. (**e**) Western blot of total and phospho-CRKL expression in the murine cell lines and the genetically manipulated 393P cell lines. (**f**) Luciferase reporter assay in H157 cells using hRL_3′ CRKL wt and mutant constructs. *p < 0.05, **p < 0.03, ***p < 0.003. The CRKL 3′UTR contains two predicted miR-200b/c/429 sites at the indicated locations. Two point mutations were introduced in each miR-200 seed sequence (red). Four wt or mutant 3′UTR constructs were individually generated and cloned into the hRL vector. (**g**) qRT-PCR analysis of CRKL after transfection of H157 cells with CRKL siRNA SMARTpool. (**h**) Migration (black) and invasion (grey) of H157 cells using CRKL siRNA SMARTpool. (**i**) H157 transfected with CRKL siRNA (or control siRNA) grown in 3D Matrigel/Collagen (1.5 mg/ml) for 9 days. Scale bar is 200 μm. (**j**) Immunofluorescence staining of transfected H157 stained for p-Src Y^418^ or p-FAK Y^861^. An average of 30 cells was counted for positive staining of their focal adhesions (FA). Data are presented as number of staining FA per cell. Scale bar is 20 μm.

Because neither Itgβ1 nor FAK are predicted miR-200 family targets and neither correlated with the EMT status of the cells, we analyzed the pan-cancer TCGA datasets^34^ and a separate compendium of publically-available lung cancer datasets^25^ for genes with negative correlation to miR-200 family expression, high correlation to ZEB1, and predicted miR200b sites in the 3′ UTR by three different prediction algorithms (Fig. 4a and Supplemental Table 2). When this list was limited to the genes associated with poor patient prognosis in lung cancer, only 29 genes came up as statistically significant, with *CRKL* as the gene with the most reasonable relationship to the observed phenotype of cytoskeletal re-organization and FAK signaling (Fig. 4a). Additionally, across the TCGA datasets (n = 9105 tumor specimens), *CRKL* levels negatively correlated with the miR-200 family levels, positively correlated with the expression of ZEB1, an EMT gene expression signature, and the p-Src Y416 levels (Fig. 4b). Finally, higher *CRKL* levels were associated with shorter overall survival, both in a compendium of lung cancer cases (Supp. Fig. 3a) and in the pan-cancer analysis of 9105 specimens from TCGA datasets (Fig. 4c).

Due to their importance in coupling signals from the ECM to intracellular signaling pathways, integrin adaptor proteins have been implicated in the phenotype of many cancer types. We further screened a candidate list of known focal adhesion complex adaptor molecules by qRT-PCR to identify mediators whose expression might regulate the Itgβ1/FAK pathway. Two isogenic cell line pairs (344SQ_vector vs 344SQ_miR-200 and 393P_vector vs 393P_Zeb1) were used to measure the expression levels of six candidates, which again identified *CRKL* in strong relationship with Zeb1 and miR-200 levels (Fig. 4d and Supp. Fig. 3b). The combined bioinformatics and experimental data identified CRKL as a candidate mediator of the phenotype. CRKL is a frequently amplified oncogene in NSCLC^28^,^35^ and an integrin adaptor molecule that plays an important role as a scaffold protein, leading to integrin-dependent complex formation, especially at sites of focal adhesions.

The protein expression and activation state of CRKL in the genetically modified 393P cells and the broader murine cell line panel, as stratified by EMT status, confirmed a potential role in coupling the ECM-integrin signals to the observed FAK pathway activation (Fig. 4e). CRKL contains two predicted target sites in the 3′ untranslated region (3′ UTR) for miR-200b/c, one with a P_CT_ of 0.29 and the other of 0.91^36^. To test whether total CRKL levels are regulated by miR-200, and could therefore modulate signaling downstream of Itgβ1, we constructed a luciferase reporter containing the wild-type CRKL 3′ UTR. The luciferase reporter assay confirmed that wild-type CRKL is a direct miR-200b/c target. Mutation of the first predicted site had no effect, while mutation of the second predicted seed sequence or both together reversed the effect of pre-miR binding (Fig. 4f). Functionally, siRNA knock-down of CRKL in H157 cells (Fig. 4g) produced a more rounded cellular morphology on fibronectin in an siRNA concentration-dependent manner (Supp Fig. S3d), reduced *in vitro* migration and invasion in Transwell assays (Fig. 4h) and 3D Matrigel/collagen I cultures (Fig. 4i). Consistent with its role as an adaptor molecule, CRKL knockdown suppressed FAK and Src localization to focal adhesion sites (Fig. 4j), but the transient knockdown approach had a blunted effect on FAK/Src activation (Supp Fig. S3c).

### EMT-dependent CRKL signaling regulates focal adhesion formation, invasion, and *in vivo* metastasis

**Figure 5:**
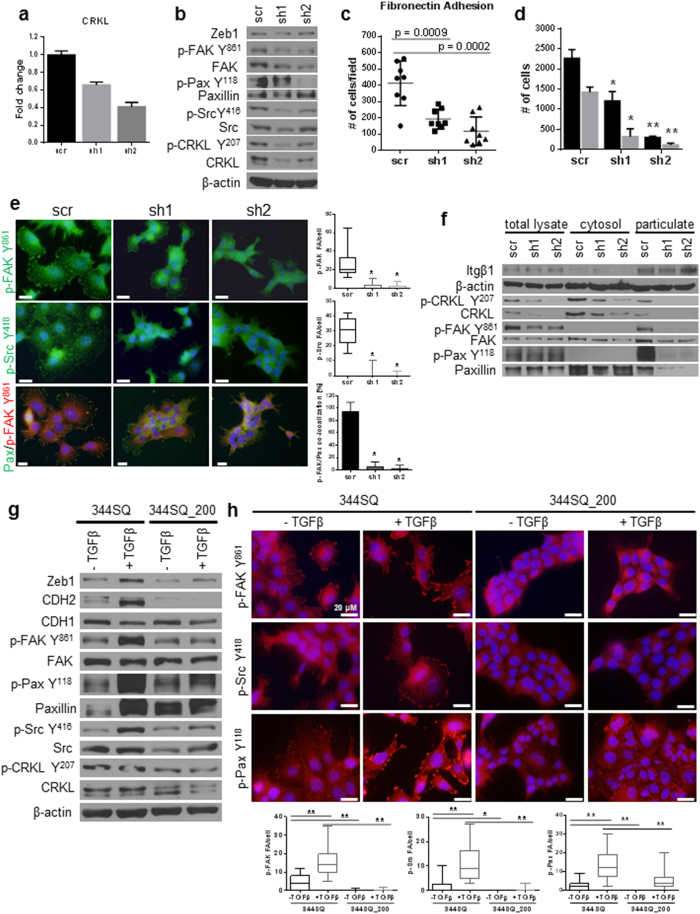
CRKL regulates FAK/Src complex formation at focal adhesions and the invasive/metastatic phenotype. (**a**) qRT-PCR and Western blot (**b**) analysis of 344SQ_shCRKL knockdown cells showed a decrease in FAK signaling. (**c**) CRKL knockdown decreased adhesion to fibronectin and 2D Transwell migration/invasion (**d**). (**e**) Immunofluorescent staining and biochemical fractionation (**f**) of CRKL knockdown cells for activated Src Y^418^, CRKL Y^207^, PaxY^118^, and p-FAK Y^861^ in the focal adhesion complex at the membrane. An average of 20–30 cells was counted for the presence of focal adhesions and data are presented per cell. Scale bar is 200 μm. *p < 0.005, **p ≤ 0.0001 (**g**) Western Blot analysis of the 344SQ cells vs. 344SQ_200 cells after a 48 hr TGFβ treatment shows an increased focal adhesion activation which is blunted with stable expression of miR-200. **(h)** Immunofluorescent staining for focal adhesion markers p-FAK Y^861^, p-Src Y^418^ and p-Paxillin Y^118^ of the cells from panel (**g**) and the quantification of the individual markers per cell. An average of 20–40 cells was counted for focal adhesions/cell with individual puncta considered a focal adhesion. Scale bar is 200 μm. *p < 0.004, **p < 0.0001.

To test the role of CRKL in EMT-mediated Itgβ1-FAK pathway activation, we used a stable shRNA knockdown approach in the mesenchymal and metastatic 344SQ murine cells with high basal levels of Zeb1. As confirmed by mRNA and protein levels, CRKL knockdown suppressed FAK pathway activation (Fig. 5a,b) without any alteration in the levels of EMT markers (Supp Fig. S3g), significantly decreased fibronectin adhesion (Fig. 5c and Supp Fig. S3e), and 2D Transwell migration/invasion (Fig. 5d). Similar to the results obtained with the CRKL siRNA in the H157 cells, CRKL knockdown in the mesenchymal murine 344SQ cells suppressed the localization of activated focal adhesion complexes at the membrane, as shown first by immunofluorescent staining for activated Src (p-Src Y^418^), FAK (p-FAK Y^861^) and co-staining for total paxillin (Fig. 5e), and second, by cellular fractionation for p-FAK Y^861^, p-CRKL Y^207^, and p-Pax Y^118^ (Fig. 5f).

We wanted to further assess the importance of repression of this particular pathway as a key mediator of miR-200 action. Based upon our previously published findings that the KP tumors have significantly elevated levels of TGFβ as a driver of EMT and metastasis^30^ and that metastatic cell lines from the model can be alternately shifted in their phenotype by miR-200 expression (Supp Fig. S3h) or treatment with TGFβ, we probed the effect of TGFβ-induced EMT on the 344SQ murine cells and demonstrated robust up-regulation of signaling through the FAK/CRKL pathway (Fig. 5g). As observed with the constitutive expression of Zeb1, this TGFβ-induced EMT was concordant with enhanced FAK (p-FAK Y^861^), Src (p-Src Y^418^) and paxillin (p-Pax Y^118^) dependent focal adhesion formation, as assayed by immunofluorescent staining (Fig. 5h). The morphologic changes, signaling activation and focal adhesion formation were completely abrogated by constitutive miR-200 expression in the cells (Fig. 5g,h and Supp Fig. S3i).

**Figure 6:**
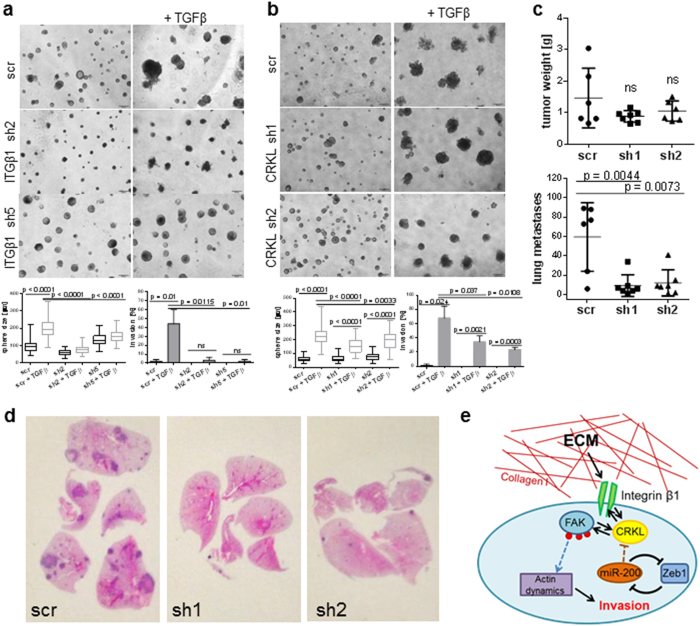
ITGβ1 and CRKL knockdown suppress the TGFβ-induced cell invasion and inhibition of CRKL reduces metastases *in vivo*. 344SQ cells with ITGβ1 (**a**) or CRKL (**b**) knockdown grown in 3D Matrigel and treated with TGFβ (treatment started at day 6). Pictures were taken at day 10. For each condition, 30 structures were measured in size and 90 structures scored for invasive protrusions. Scale bar represents 200 μm. (**c**) Primary tumor weight and lung metastases in mice injected with control or 344SQ_shCRKL knockdown cells (top graphs), and as visualized by the H&E staining of lung sections (**d**). (**e**) Proposed model of the interaction of ITGβ1-collagen I leading to activation of FAK regulated by the mir-200 target CRKL to produce downstream invasion and metastasis.

To extend the importance of the Itgβ1/CRKL signaling pathway in mediating TGFβ-induced invasion with EMT, we used the 344SQ cells with stable shRNA-based knockdown of Itgβ1 or CRKL in 3D assays (Fig. 6a,b). Either Itgβ1 or CRKL knockdown repressed TGFβ-induced invasion, although knockdown of Itgβ1 had the stronger effect, potentially due to its more complete knockdown in these cells. Given the ability of CRKL knockdown to repress FAK pathway signaling, *in vitro* migration and invasion, focal adhesion formation and the effects of TGFβ stimulation, we assessed whether the 344SQ CRKL shRNA cells could grow and metastasize *in vivo*. Testing by implantation into syngeneic mice produced similar primary tumor growth to control scramble knockdown cells, but strongly suppressed the number and size of distant lung metastases (Fig. 6c,d), as seen in the gross and H&E stained lung sections (Supp Fig. S3f and Fig. 6d).

Based upon the multiple lines of *in vitro* and *in vivo* data included herein, a schema is presented that outlines our working model for the role of miR-200 in regulating tumor cell activation by interactions with the ECM (Fig. 5j). Either intrinsic dysregulation of Zeb1 or TGFβ-mediated suppression of miR-200 can de-repress CRKL expression to drive subsequent collagen I-mediated ITGβ1-FAK signaling.

## Discussion

The complex, multi-step metastatic process requires alteration of multiple complementary cellular functions to produce invasion, migration through the blood or lymphatic systems, extravasation at distant sites and colonization. The epithelial-mesenchymal transition has been posited as a developmental program coopted during tumor development that can facilitate many of the required steps. As a master EMT regulator, the Zeb1-miR-200 double-negative feedback loop has been shown by multiple groups to play a prominent role in tumor invasion and metastasis. However, the full complement of cellular functions altered during EMT that are specifically regulated by gain of Zeb1 expression and loss of miR-200 is unclear. Using the KP model system and human lung cancer cell line models, we have addressed this issue.

Invasion into and through the underlying basement membrane is critical to epithelial tumor growth and metastasis. Our prior work with KP cells in synthetic matrix systems demonstrated the ability to recapitulate normal epithelial morphogenesis^24^, but despite induction of EMT by TGFβ treatment or matrix stiffness, invasion or migration through these matrices was suppressed. Similarly, the 3D culture model data presented here demonstrate that cell-autonomous tumor cell changes are only sufficient to produce a hyper-proliferative phenotype, but insufficient to produce invasion into a laminin-rich Matrigel. In contrast, concomitant changes in the miR-200 levels and manipulation of the matrix composition by inclusion of type I collagen produced robust invasion. Our extensive prior bioinformatic analyses incorporating mRNA and proteomic profiles of tumor cells with high/low miR-200 expression revealed genome-wide changes altering the balance of cell-cell and cell-matrix interactions, along with substantial effects on the surrounding ECM composition^23^. These findings suggest that during tumor progression miR-200 loss controls cell-intrinsic EMT features and coordinates complementary changes in the surrounding tumor microenvironment. Our demonstration here that β1-integrin-collagen I interactions are critical to the invasive phenotype in 3D culture fully supports the broader proteomic profiling data and a dynamic role for type I collagen, which can undergo crosslinking to produce ECM stiffening associated with tumor growth and progression^37^,^38^. Conversely, the ability of miR-200 expression to suppress cellular response to the ECM also explains the normalizing effects of laminin-rich matrices on tumor cells^30^, a counter-balancing effect that enhances tumor cell adaptability to the microenvironment and facilitates the later steps in metastasis, such as distant colonization.

Tumors are remarkably heterogeneous and our findings argue that similarly activated cells differentially interacting in the tumor interior versus the surrounding ECM produce differential biochemical activation of tumor cell subpopulations. Biochemically, the increased matrix responsiveness of tumor cells upon Zeb1 expression was due to enhanced FAK signaling, which was essentially shutoff with high miR-200 levels and phenocopied by CRKL or Itgβ1 knockdown. Consistently, the importance of miR-200 in modulating CRKL regulation of collagen I-Itgβ1-dependent cell signaling was emphasized by results from multiple different experimental systems, including 2D and 3D *in vitro* assays with murine and human cells, knockdown-based strategies at several points in the pathway, and *in vivo* metastasis driven by loss of miR-200 expression in the syngeneic KP model.

Given the remarkable pleiotropy of the miR-200 family in regulating genome-wide targets to suppress EMT, the regulation of specific cellular functions is still being elucidated. Recent studies have identified several actin-associated genes as targets, such as moesin, FHOD1, and PPM1F^39^,^40^. Here we have shown that the adaptor molecule, CRKL, is a direct miR-200bc target, functionally affecting experimental migration, invasion and metastasis of lung cancer cells and clinically prognostic of outcome in multiple tumor types from analysis of TCGA datasets. Our findings have identified CRKL as critical to integrin-dependent activation of the cancer cells and localization of activated FAK and Src to focal adhesions. In this manner, direct regulation by miR-200 of the CRKL adaptor suppresses FAK/Src complex formation at the membrane and subsequent downstream signaling from collagen I-Itgβ1. Furthermore, our data suggest that CRKL not only enhances the outside-in signaling through Itgβ1, but is also necessary for the inside-out signaling required to maintain the cell-matrix contacts critical to continuous invasion. A very recent study from the Goodall lab^41^ using breast cancer cells to identify transcriptome-wide miR-200 targets by an Ago-HITS-CLIP and sequencing approach identified multiple genes involved in invadopodia formation, MMP activity, and regulation of actin cytoskeletal dynamics. The work presented here is consistent with their findings, describes a specific requirement for ECM-mediated tumor cell activation and demonstrates the importance of this pathway in tumor cell EMT, *in vivo* metastatic spread, and clinical outcome.

## Materials and Methods

### Animal studies

All animal experiments were approved by the M.D. Anderson Cancer Center IACUC and performed in accordance to their guidelines. Wild-type male and female 129/sv mice of a minimum 2 months old were used. 0.5–1 × 10^6^ cells were subcutaneously injected in the flank in 100 μl serum-free RPMI. The animals were monitored for tumor burden and sacrificed once the tumor size exceeded 15 mm or developed ulceration. Mice were examined for metastasis and tissues from the primary subcutaneous tumor, lungs and any organs with visible metastases were collected. The results are represented as mean ± standard deviation and student’s *t*-test was performed for statistical significance.

### Cell culture

As previously described^30^, cell lines derived from the mutant Kras/p53 mouse model and human lung cancer cells H157 were cultured in RPMI1640 with 10% fetal bovine serum (FBS). The generation of the genetically modified 393P and 344SQ cell lines stably expressing ZEB1 or the miR-200b, −200a, −429 cluster, respectively, are described in previous publications^30^,^42^, while the H157 cells with doxycycline-inducible expression of miR-200a, −200b or the combination were previously described in^32^ and^43^. All blocking antibodies and Ig controls were purchased from BD Pharmingen and used at a final concentration of 8 μg/ml: Itgβ1 (BD 555002), Itgβ3 (BD 553343), IgM (BD 553957), IgG (553950). Lentiviral-based Itgβ1 shRNA were purchased from Thermo Scientific (mouse: TRCN0000066643, TRCN0000066644, TRCN0000066645, TRCN0000066646, TRCN0000066647 (also targets human Itgβ1), human: TRCN0000029645, TRCN0000029648). The human CRKL siRNA SMARTpool was purchased from Dharmacon (L-012023-00-0005) and used at a final concentration of 25 nM. Lentiviral-based CRKL shRNA were purchased from Thermo Scientific (mouse: TRCN0000097199, TRCN0000097200, TRCN0000097202, TRCN0000097203). siRNA transfections were done using DharmaFECT I (Dharmacon), shRNA transfections were done using Lipofectamine® LTX (Invitrogen) and PLUS™ reagent (Invitrogen). Pre-miRs were purchased from Ambion and transfected at a final concentration of 30 nM using Lipofectamine® 2000 (Invitrogen). The final DNA concentration used in the Luciferase Reporter Assays was 500 ng. TGFβ treatment (CS8915LF, 5 ng/ml) was carried out for 48 hrs before lysates were collected or cells used for immunofluorescence.

### Migration and Invasion Assays

Cells were seeded at 5 × 10^4^ per well in serum-free media in a 24-well Transwell or Matrigel plate (BD Biosciences, pore size 8 μm). RPMI with 10% FBS was placed in the lower chamber as chemoattractant and cells were allowed to migrate for 6 (H157 cells) or 16 hrs (murine cells) at 37 °C, 5% CO2. The migrated/invaded cells were stained with 0.1% crystal violet, captured in five microscopic fields at 4x magnification per well and counted. The results are represented as mean ± standard deviation and student’s *t*-test was performed for statistical significance. The graphs in each figure represent one experiment. Each assay was performed in triplicate.

### Cell Adhesion Assay

Wells of a 24-well plate or 12 mm glass coverslips were coated with a thin layer of fibronectin (10 μg/ml, Sigma F1141). After the incubation time, each well was washed twice with PBS to remove unattached cells, fixed for 10 min at room temperature with 10% formalin, washed with PBS and stained using 0.1% Crystal Violet/PBS or stained with DAPI for the nucleus. Images are representatives of the triplicates performed for each condition.

### Quantitative Real Time PCR

RNA was isolated using TRIzol® Reagent (Invitrogen) according to the manufacturer’s protocol and reverse transcribed into cDNA using qScript™ Reagent (Quanta Biosciences). mRNA levels were measured as previously described^30^. Primer sequences are listed in Supplementary Table 1.

### Western Blot Analysis

Cell lysates were prepared according to the RIPA buffer protocol (CS9806). Antibodies were obtained from the following companies: Zeb1 (sc-25388), N-cadherin (BD610921), E-cadherin (BD610182), Vimentin (CS3932), p-Src Y416 (CS2101), Src (CS2108), p-FAK Y861 (Invitrogen 44-626G), FAK (Invitrogen AHO0502), p-Paxillin Y118 (Abcam4833), Paxillin (Abcam2264), Itgβ1 (CS4706), p-p130Cas Y410 (CS4011), p130Cas (MP06-500), p-CRKL Y207 (CS3181), CRKL (MP05-414).

### 3D culture

Cells were grown in 8-well chamber slides coated with Matrigel (BD 356231) or Matrigel/collagen I (BD 354249, at the indicated concentration), as previously described^30^. The structure sizes and invasion were scored at the end of the experiment, with structures counted invasion-positive if  ≥1 protrusions were present. Immunofluorescent staining of the 3D cultures was performed as described^30^. TGFβ (5 ng/ml) was added at day 6 or 7 and the media was replaced every 48 hrs.

### Immunofluorescence

Acid-washed 12 mm coverslips were coated with fibronectin (10 μg/ml, Sigma F1141) before 15,000-20,000 cells were plated and grown overnight. Cells were fixed with 4% paraformaldehyde, then permeabilized with 0.1% Triton X-100. Staining of F-actin was performed using Alexa Fluor 546 Phalloidin and DAPI for the nuclear stain contained in the mounting solution. The slides were incubated with the primary antibodies overnight at 4 °C (p-Src Y^418^, MP569373, 1:50; p-FAK Y^861 ^Invitrogen 44626, 1:50; Paxillin BD610620, 1:100; p-Paxillin Y^118^, ab4833, 1:50). Quantification of antibody staining per focal adhesion was done on an average of 15–30 cells in which individual puncta were considered a focal adhesion. The graph shows positive focal adhesion staining/cell. Student’s *t*-test was performed for statistical significance.

### Luciferase Reporter Assay

3′-UTRs were amplified by PCR from genomic DNA and mutants generated using a QuikChange® II XL site-directed mutagenesis kit (Stratagene), which were subsequently cloned into the hRL vector. One day before transfection, 3 × 10^4^ cells were seeded in 24-well plates and co-transfected with 50 ng pGL3 control vector and 500 ng hRL constructs. Pre-miRs were added at a final concentration of 30 nM (Ambion). After 48 hrs luciferase activity was measured.

### Cytosol/particulate separation

The separation of cytosol and particulate was performed using a cytosol/particulate rapid separation kit per the manufacturer’s instructions (BioVision #K267-50). Itgβ1 is used as a loading control for the particulate fraction, β-actin for the total lysate and the cytosolic fraction.

### FACS analysis

Cells were stained with either control (PE Hamster IgG BD 553965, 1 μg per 1 × 10^6^ cells) or CD29 antibody (PE Hamster CD29 BD 562801, 1 μg per 1 × 10^6^ cells) for 45 min. PE positive cells were analyzed by the Flow Cytometry and Cellular Imaging Core Facility at MD Anderson Cancer Center.

### Proliferation Assay

One day prior to the assay 750 cells/96 well were plated in replicates of 8. To measure proliferation, WST-1 reagent was used according to the manufacturer’s protocol. The absorbance was measured at 450 nM 1 hour after adding the WST-1 reagent over a period of 4 days. Multiple *t*-test was used for statistical significance.

### Analysis of human tumor molecular datasets

For analysis of *CRKL* mRNA expression and cancer patient survival, we examined a previously-assembled compendium dataset^25^ of 11 published expression profiling datasets for human lung adenocarcinomas (n = 1,492 tumors). In addition, we collected molecular data on 9,617 tumors of various histological subtypes (ACC project, n = 79; BLCA, n = 408; BRCA, n = 1094; CESC, n = 304; CHOL, n = 36; COAD/READ, n = 625; DLBC, n = 28; GBM, n = 161; HNSC, n = 520; KICH, n = 66; KIRC, n = 533; KIRP, n = 290; LAML, n = 173; LGG, n = 516; LIHC, n = 371; LUAD, n = 515; LUSC, n = 501; MESO, n = 86; OV, n = 263; PAAD, n = 178; PCPG, n = 179; PRAD, n = 497; SARC, n = 259; SKCM, n = 468; TGCT, n = 150; THCA, n = 501; THYM, n = 120; UCEC, n = 545; UCS, n = 57; UVM, n = 80) from TCGA^34^, for which RNA-seq data (v2 platform) were available. A subset of the 9,617 TCGA pan-cancer set also had survival data and data on other molecular platforms, as indicated (Fig. 4a–c). TCGA RPPA data were from Akbani *et al.*^44^. Patient survival was capped at 200 months. Pearson’s correlation between features was computed using log-transformed expression values. EMT signature score across human tumors was computed as previously described^45^.

## Additional Information

**How to cite this article**: Ungewiss, C. *et al.* The microRNA-200/Zeb1 axis regulates ECM-dependent β1-integrin/FAK signaling, cancer cell invasion and metastasis through CRKL. *Sci. Rep.* **6**, 18652; doi: 10.1038/srep18652 (2016).

## Supplementary Material

Supplementary Information

Supplementary Table S2
