# Supplementary material for: The microRNA-200/Zeb1 axis regulates ECM-dependent β1-integrin/FAK signaling, cancer cell invasion and metastasis through CRKL
Source: Sci Rep. 2016 Jan 5;6:18652. doi: 10.1038/srep18652 (PMC4700473; doi:10.1038/srep18652)
Supplement: Supplementary Information [file srep18652-s1.pdf]

The microRNA-200/Zeb1 axis regulates ECM-dependent β1-integrin/FAK signaling, cancer cell invasion and metastasis through CRKL

Christin Ungewiss[1], Zain H. Rizvi[1], Jonathon D. Roybal[1], David Peng[1], Kathryn Gold[1], Dong-Hoon Shin[1], Chad J. Creighton[3,4], Don L. Gibbons[1,2,5]

[1]Department of Thoracic/Head and Neck Medical Oncology, The University of Texas MD Anderson Cancer Center, Houston, TX 77030, USA
[2]Department of Molecular and Cellular Oncology, The University of Texas MD Anderson Cancer Center, Houston, TX 77030, USA.
[3]Department of Bioinformatics and Computational Biology, 1400 Pressler St., The University of Texas MD Anderson Cancer Center, Houston, TX 77030
[4]Department of Medicine and Dan L. Duncan Cancer Center, Baylor College of Medicine, Houston, TX 77030
[5]Corresponding author: dlgibbon@mdanderson.org, 713-792-9536 (phone), 713-792-1220 (fax).

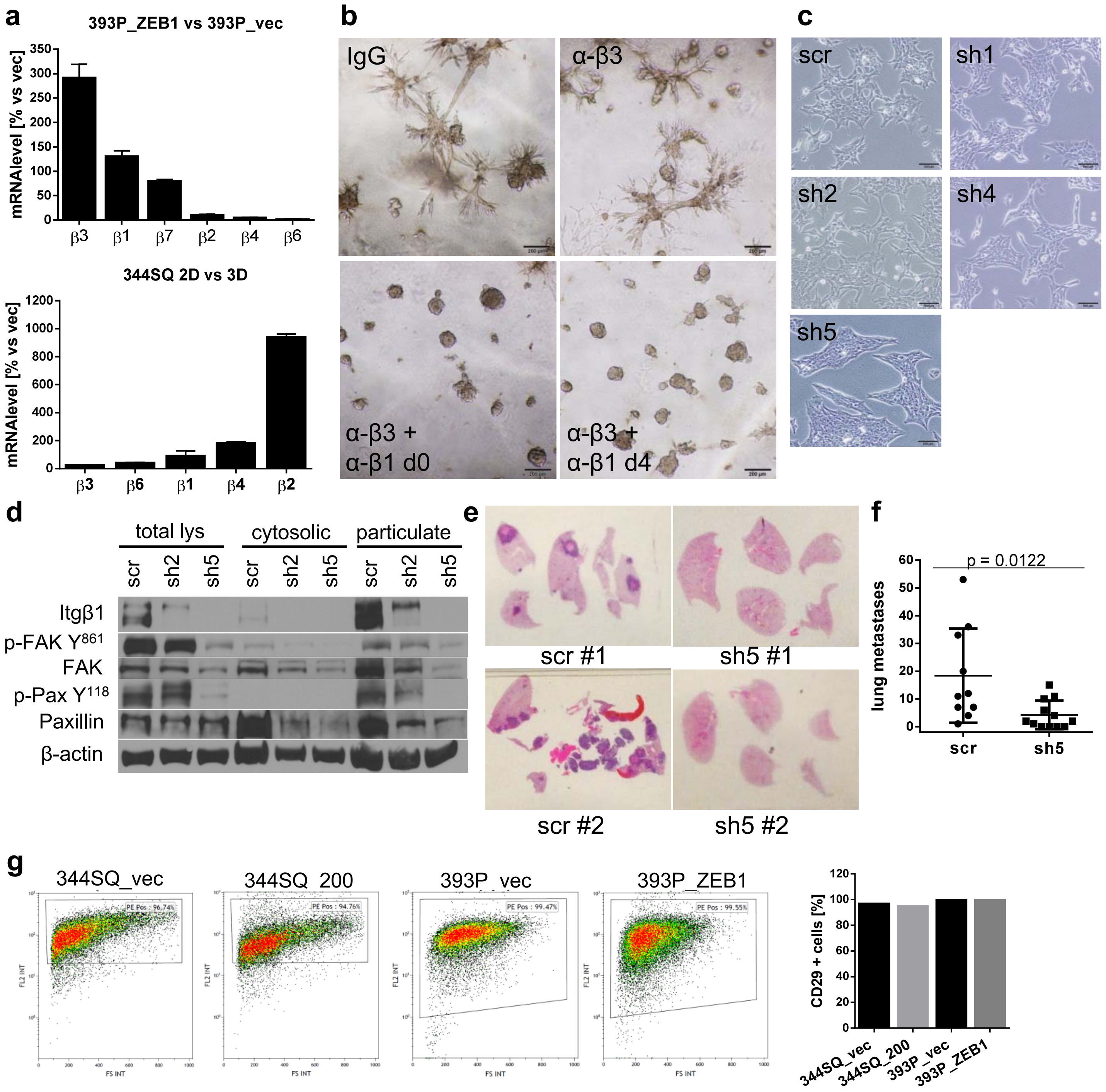

Supplementary Figure S1. Integrin β1 is required for mesenchymal cell invasion. (A) Quantitative RT-PCR analysis of Integrin β subunits in 393P_ZEB1 vs vec cells (top panel) and in 344SQ grown in 2D vs 3D Matrigel (lower panel) (B) 393P_ZEB1 cells grown in 1.75 mg/ml Matrigel/Collagen I and treated with an Integrin β3-blocking antibody alone, in combination with an Integrin β1-blocking antibody (added at the days indicated) or IgG control for 7 days. (C) Morphology of 393P_ZEB1 cells after ITGβ1 knockdown. (D) Western Blot analysis of 344SQ ITGβ1 knockdown cells after cytosolic and particulate separation. (E) H&E of lung sections of animals injected with 344SQ scr or 344SQ ITGβ1 sh5. (F) ITGβ1 knockdown in 344SQ cells reduces lung metastases *in vivo* (with one animal outlier removed).(G) FACS analysis of the murine cells for PE-CD29 positive cells.

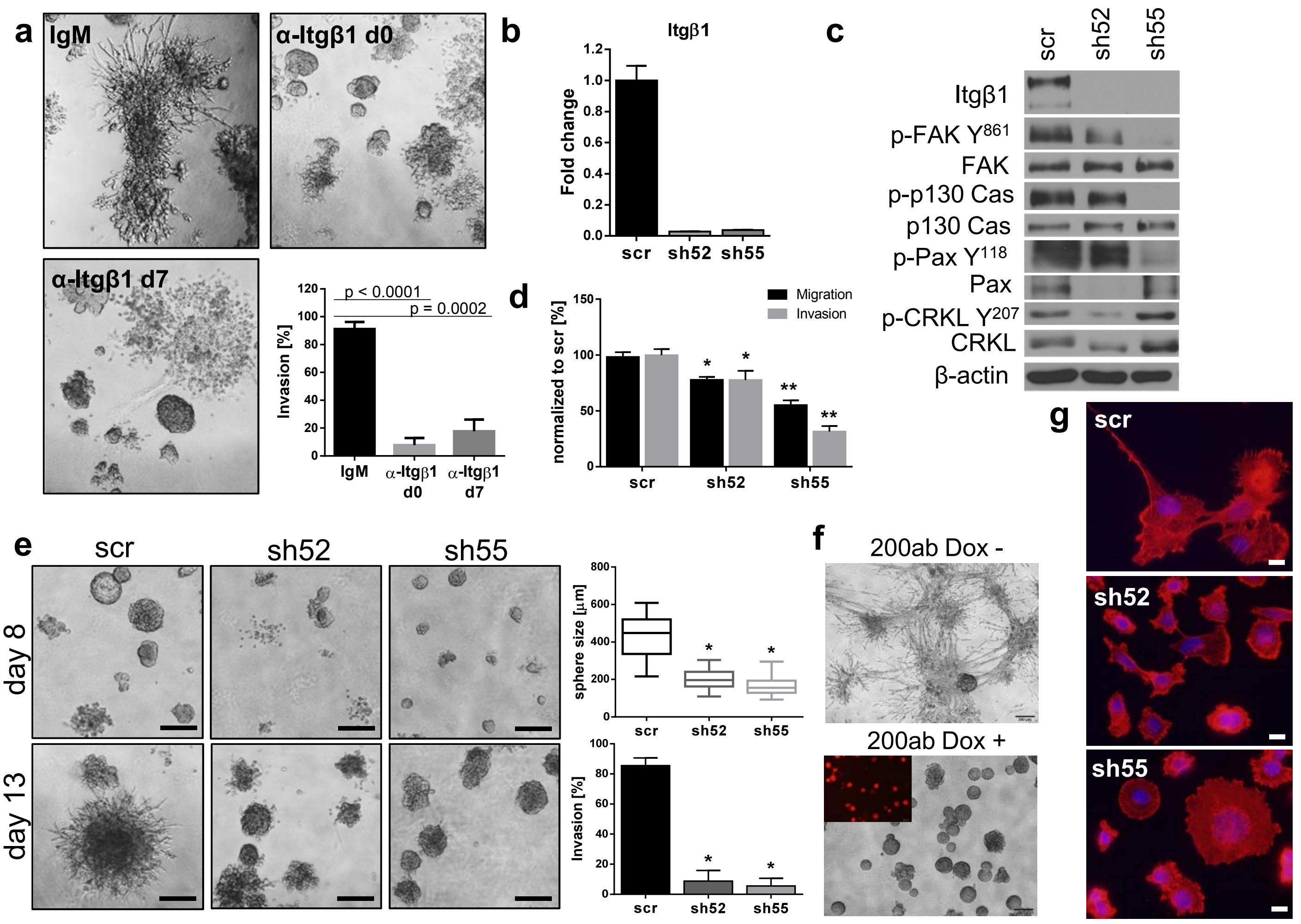

Supplementary Figure S2. Integrin β1-collagen I contact is necessary for H157 cell growth and invasion. (A) H157 cells grown in 1. 5 mg/ml Matrigel/Collagen I and treated with an ITGβ1-blocking antibody from day 0 or day 7 or IgM control. 90 structures were scored for invasiveness. (B) Quantitative RT-PCR and (C) Western Blot analysis of H157 cells after Itgβ1-shRNA knockdown shows a partial MET. (D) ITGβ1-knockdown cells grown on Matrigel/Collagen I for 13 days and analyzed for sphere size (30 structures) and invasiveness (90 structures). Scale bar is 200 μm. * $p \leq 0.0001$ (E) In vitro migration and assay for the Itgβ1 shRNA cells. * $p < 0.001$, ** $p < 0.0001$ (F) H157 cells grown in 3D culture (Matrigel/Collagen mixture) for 11 days. (G) H157 Itgβ1 knockdown cells stained for F-actin with Phalloidin and DAPI for the nucleus. Scale bar is 200 μm.

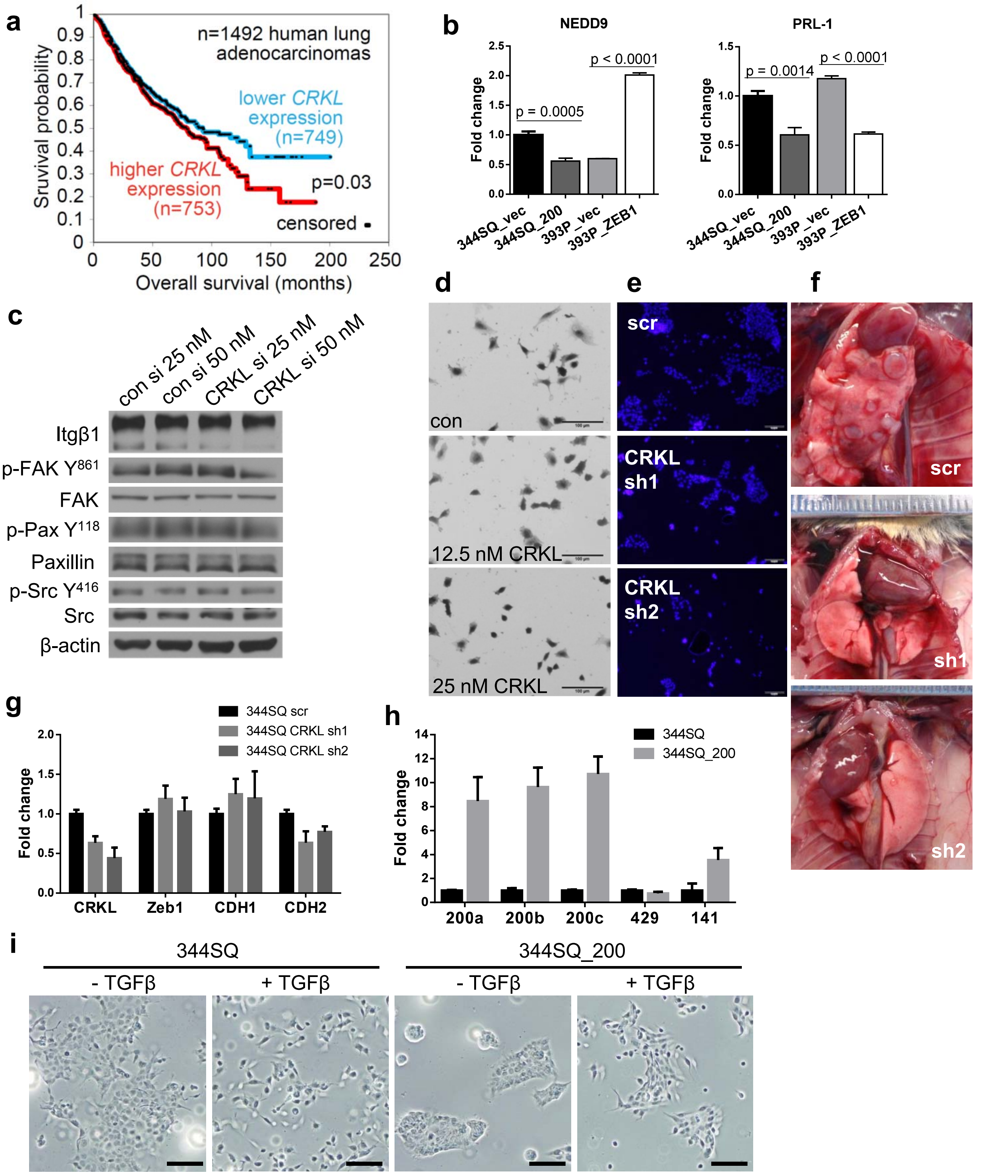

Supplementary Figure S3. CRKL knockdown causes a decrease in cell adhesion and *in vivo* metastases. (A) KM survival analysis of patients from the lung compendium database stratified based upon CRKL expression. (B) qRT-PCR analysis of adaptor molecules in the paired epithelial and mesenchymal murine cell lines. (C) Western Blot analysis of CRKL siRNA 48 hrs post-transfection using different siRNA concentrations. (D) Adhesion assay of H157 on Fibronectin transfected with CRKL siRNA. (E Adhesion assay of 344SQ CRKL knockdown cells on Fibronectin stained with DAPI. (F) Images of the lungs of mice injected with 344SQ scr or CRKL knockdown cells. (G) qPCR analysis of the EMT markers in the 344SQ CRKL shRNA cells. (H) miR-200 expression levels in the 344SQ vs. 344SQ_200 cells. (i) Brightfield images of the 344SQ vs. 344SQ_200 cells treated with TGFβ for 48 hrs on Fibronectin coated coverslips. Scale bar is 100 μm.

Supplementary Table 1.

qPCR primer 5' to 3'

ms L32: F	GGAGAAGGTTCAAGGGCCAG
ms L32: R	TGCTCCCATAACCGATGTTG
ms Zeb1: F	ATGCTCTGAACGCGCAGC
ms Zeb1: R	AATCGGCGATCTTTGAGAGCT
ms CDH1: F	CCATCTCAAGCTCGCGGATA
ms CDH1: R	TCCAACGTGGTCACCTGGT
ms CDH2: F	TCCAGAGGGATCAAAGCCTGGGAC
ms CDH2: R	CCGCATCAATGGCAGTGACCGT
ms Vim: F	TCCAAGCCTGACCTCACTGC
ms Vim: R	TTCATACTGCTGGCGCACAT
ms Crb3: F	CGGACCCTTTCACAAATAGCA
ms Crb3: R	CGTTGGACTCATCACCTGGG
ms Itgb1: F	CTACTTCTGCACGATGTGATGAT
ms Itgb1: R	TTGGCTGGCAACCCTTCTTT
ms Itgb2: F	CAGGAATGCACCAAGTACAAAGT
ms Itgb2: R	CCTGGTCCAGTGAAGTTCAGC
ms Itgb3: F	CCACACGAGGCGTGAACTC
ms Itgb3: R	CTTCAGGTTACATCGGGGTGA
ms Itgb4: F	AGAGCTGTACCGAGTGCATC
ms Itgb4: R	TGGTGTCGATCTGGGTGTTCT
ms Itgb6: F	GAAAACCCTGTCTCCCGCATA
ms Itgb6: R	CGCTGAGAGGCTTATTTTGAAGG
ms Itgb7: F	ACCTGAGCTACTCAATGAAGGA
ms Itgb7: R	CACCGTTTTGTCCACGAAGG
ms CRKL:F	CCTGGACACTACCACCTTAATCG
ms CRKL:R	TCTTCTGCTGTAGGTAAGTTGGG
ms CRK:F	CTAATGCCTACGACAAGACAGCC
ms CRK:R	TGGGAAGTGACCTCGTTTGCCA
ms PINCH: F	GGGTTTGTCAAGAATGCTGGCAG
ms PINCH: R	GCACAGTTGAAGTGGTCTGGATG
ms ILK: F	GTGCTGAAGGTTCGTGACTGGA
ms ILK: R	TCCAGTGTGTGATGAGGGTTGG
hs L32: F	CCTTGTGAAGCCCAAGATCG
hs L32: R	TGCCGGATGAACTTCTTGGT
hs Zeb1: F	GGCATACACCTACTCAACTACGG
hs Zeb1: R	TGGGCGGTGTAGAATCAGAGTC
hs Itgb1: F	GGATTCTCCAGAAGGTGGTTTCG
hs Itgb1: R	TGCCACCAAGTTTCCCATCTCC
hs CRKL: F	ATCCACTACCTGGACACCACCA
hs CRKL: R	CCAGGTTATCTTCTGCTGTAGGC
